# Genome Instability-Derived Genes Are Novel Prognostic Biomarkers for Triple-Negative Breast Cancer

**DOI:** 10.3389/fcell.2021.701073

**Published:** 2021-07-12

**Authors:** Maoni Guo, San Ming Wang

**Affiliations:** Cancer Centre and Institute of Translational Medicine, Faculty of Health Sciences, University of Macau, Macau, China

**Keywords:** TNBC, mutation, copy number variation, genome instability, prognosis

## Abstract

**Background:**

Triple-negative breast cancer (TNBC) is an aggressive disease. Recent studies have identified genome instability-derived genes for patient outcomes. However, most of the studies mainly focused on only one or a few genome instability-related genes. Prognostic potential and clinical significance of genome instability-associated genes in TNBC have not been well explored.

**Methods:**

In this study, we developed a computational approach to identify TNBC prognostic signature. It consisted of (1) using somatic mutations and copy number variations (CNVs) in TNBC to build a binary matrix and identifying the top and bottom 25% mutated samples, (2) comparing the gene expression between the top and bottom 25% samples to identify genome instability-related genes, and (3) performing univariate Cox proportional hazards regression analysis to identify survival-associated gene signature, and Kaplan–Meier, log-rank test, and multivariate Cox regression analyses to obtain overall survival (OS) information for TNBC outcome prediction.

**Results:**

From the identified 111 genome instability-related genes, we extracted a genome instability-derived gene signature (GIGenSig) of 11 genes. Through survival analysis, we were able to classify TNBC cases into high- and low-risk groups by the signature in the training dataset (log-rank test *p* = 2.66e−04), validated its prognostic performance in the testing (log-rank test *p* = 2.45e−02) and Molecular Taxonomy of Breast Cancer International Consortium (METABRIC) (log-rank test *p* = 2.57e−05) datasets, and further validated the predictive power of the signature in five independent datasets.

**Conclusion:**

The identified novel signature provides a better understanding of genome instability in TNBC and can be applied as prognostic markers for clinical TNBC management.

## Introduction

Triple-negative breast cancer (TNBC) accounts for ∼15% of all breast cancer cases and is characterized by the absence of estrogen receptor (ER), progesterone receptor (PR), and epidermal growth factor receptor 2 (HER2) (Oakman et al., 2010). TNBC often occurs at a young age, highly aggressive, and metastatic with a poor prognosis (Haffty et al., 2006; Dent et al., 2007). A part of TNBC is related with the germline mutation in *BRCA1* and *BRCA2*, but the cause for most of the TNBC remains unclear (Song et al., 2014). With its unusual clinic outcome, a TNBC-specific prognostic signature will be highly valuable for clinical management of TNBC cases (Mersin et al., 2008; Tan and Swain, 2008).

Genomic instability is a major hallmark in cancer and an important prognostic factor associated with cancer progression and survival (Suzuki et al., 2003; Negrini et al., 2010). Genome instability-associated signatures have been identified in certain types of cancer. For example, a genomic instability-derived three-miRNA signature was used as a risk predictor for invasive breast cancer (Bao et al., 2021); a 12-genomic instability-derived gene expression signature was identified as a clinical outcome predictor for breast cancer (Habermann et al., 2009). Genome instability-related mutation and copy number variation (CNV) were also identified in TNBC (Schmitt et al., 2012). For instance, germline mutations in *BRCA1*, *BRCA2*, *ATM*, *PALB2*, *RAD51D*, and *RAD50* disrupted DNA damage repair pathways in TNBC (Wu et al., 2019); *FOSL1* had significantly higher CNV gains in TNBC than in other types of breast cancer (Serino et al., 2019); *PIK3CA* had high mutation frequency and copy number gains and highly ethnic-specific in TNBC (Jiang et al., 2019); somatic mutation and CNV-derived genomic metrics were significantly associated with immune prognostic category in TNBC (Karn et al., 2017). Furthermore, somatic mutations and CNVs were associated with dysregulation of multiple genes in TNBC. For example, mutations in *MYH9* and *HERC2* were both associated with lower lymphocyte-specific kinase (LCK) metagene expression in TNBC (Safonov et al., 2017); *JAK2* and *PD-L1* amplifications upregulated *PD-L1* expression by disturbing the JAK/STAT1 pathway in TNBC (Chen M. et al., 2018); high expression of *PD-L1* was associated with significant *CD274* gene copy number gain in TNBC (Guo et al., 2016). However, the clinical impact of these abnormalities as prognostic markers in TNBC remains largely unclear.

We hypothesized that there could be a genome instability-derived signature involved in the tumorigenesis and development of TNBC. We further reasoned that genomic instability-derived somatic mutation and CNV in TNBC could disturb gene expression in TNBC; therefore, expression difference in TNBC could be used as prognostic markers to predict clinical outcome of TNBC.

In this study, we first calculated the accumulative counts of somatic mutation and CNV in TNBC cases and selected the top and bottom 25% of the ranked cases. We then identified 111-genomic instability-derived genes to divide the cases into genomic unstable (GU) and genomic stable (GS) groups. Furthermore, we identified a genome instability-derived gene signature (GIGenSig) of 11 genes to classify TNBC cases into high- and low-risk groups. We validated the results using multiple independent TNBC datasets. Our study provides a GIGenSig as a prognosis marker to predict the clinical outcome of TNBC.

## Materials and Methods

### Datasets Used for the Study

We downloaded the METABRIC (Molecular Taxonomy of Breast Cancer International Consortium) breast cancer datasets (Pereira et al., 2016) from the cBioPortal database^1^, including clinical information, gene expression, somatic mutation, and CNV data. The expression profile was processed as log intensity level of Illumina Human v3 microarray. We also downloaded the version 19 gene annotation file from the GENCODE database^2^. Then, we randomly divided the TNBC cases into the training dataset and the testing dataset with the same size of living and deceased overall survival (OS) status in each dataset. The training dataset consisting of 150 TNBC samples was used to identify the prognostic signature and build the prognostic risk model; the testing dataset consisting of 149 TNBC patients was used to validate the prognostic model. Clinical characteristics for the training, testing, and METABRIC datasets are summarized in Table 1. The Cancer Genome Atlas (TCGA^3^), Shanghai TNBC data^4^ (Jiang et al., 2019; Goldman et al., 2020), and three additional independent datasets of GSE21653, GSE31448, and GSE25066 from the GEO database^5^,^6^,^7^ were used to validate the performance of the prognostic risk model (Hatzis et al., 2011; Sabatier et al., 2011a,b). For TCGA and Shanghai RNAseq datasets, we downloaded or processed the gene-level transcription estimates in log2(*x* + 1) transformed RSEM normalized count. For the other three GEO microarray datasets, they were processed using the robust multichip average (RMA) algorithm for background adjustment (Irizarry et al., 2003a,b), and the Affymetrix GeneChip probe-level data were log2 transformed. The platform information for Affymetrix Human Genome U133 Plus 2.0 Array was downloaded from the Affymetrix website^8^. Gene expression data from the Affymetrix-based expression profiling were obtained by repurposing microarray probes based on the platform information and the gene annotation file from the GENCODE database (release 19, see text footnote 2).

**TABLE 1:** Clinical information for triple-negative breast cancer (TNBC) patient datasets used in this study.

**Characteristics**		**Training dataset (*N* = 150)**	**Testing dataset (*N* = 149)**	**Molecular Taxonomy of Breast Cancer International Consortium (METABRIC) dataset (*N* = 299)**	*p*-Value*
Age (%)	Age < 55	68(45.33)	75(50.34)	143(47.83)	0.687
	Age ≥ 55	82(54.67)	74(49.66)	156(52.17)	
Menopausal_status (%)	Pre	49(32.67)	58(38.93)	107(35.79)	0.529
	Post	101(67.33)	91(61.07)	192(64.21)	
Tumor stage (%)	I/II	100(66.67)	92(61.74)	192(64.21)	0.532
	III/IV	16(10.67)	9(6.04)	25(8.36)	
	Unknown	34(22.67)	48(32.21)	82(27.42)	
Grade (%)	G1/2	17(11.33)	22(14.77)	39(13.04)	0.664
	G3	132(88.00)	125(83.89)	257(85.95)	
	Unknown	1(0.67)	2(1.34)	3(1.01)	
OS_status (%)	Living	69(46.00)	69(46.31)	138(46.15)	0.999
	Deceased	81(54.00)	80(53.69)	161(53.85)	

*p-value: Chi square test.

### Identification of Genome Instability-Related Genes in TNBC

To identify genome instability-related genes in TNBC, we first processed gene expression and genomic alteration profiles. For gene expression profile, we extracted the expression log intensity levels (Illumina Human v3 microarray) for 16,331 protein-coding genes; for mutations, silent mutations were removed; for CNV profile, we only retained high-level amplification and homozygous deletion evaluated by GISTIC2 segment (Mermel et al., 2011). Then, we integrated TNBC gene expression and genomic alteration data as shown in Supplementary Figure 1: (1) extracted gene expression, somatic mutation, and CNV profiles in the 299 TNBC cases; (2) constructed a binary matrix by integrating somatic mutation and CNV profiles; (3) calculated the accumulated alterations for each case; (4) took the top 25% and the bottom 25% of alternated cases as GU group and GS group; (5) compared the gene expression between GU and GS groups by using the R package “limma”; and (6) identified genome instability-related genes with a Benjamini and Hochberg (BH) adjusted *p*-value < 0.05 and logFC (fold-change) > 1 or < −1 between GU group and GS group.

### Functional Enrichment Analysis

We applied enrichGO and enrichKEGG functions in the Bioconductor package “clusterProfiler” to identify the functions and pathways of the genome instability-related genes (Yu et al., 2012). We also performed Gene Ontology (GO) and Kyoto Encyclopedia of Genes and Genomes (KEGG) functional enrichment annotation using the Database for Annotation, Visualization and Integrated Discovery (DAVID tool^9^, version 6.8) (Huang da et al., 2009). The Benjamini *p*-adjust < 0.05 was considered as statistically significant in “clusterProfiler” and *p*-value < 0.05 for DAVID analyses.

### Statistical Analysis

To identify the genes predictive for TNBC OS, a univariate Cox proportional hazards regression analysis was performed to evaluate the relationship between the expression level of each gene and patient OS in the training dataset. Only the genes with a *p*-value < 0.05 were taken as statistically significant survival predictors. To construct a predictive model, a multivariate Cox regression model was applied for these selected genes with OS in the training dataset. A risk score formula was built to evaluate the risk of each patient to develop TNBC as follows:

$$\mathrm{Overall}\,\mathrm{risk}\,\mathrm{score}\,\left(\text{ORS}\right)=\sum_{i=1}^{N}\left(E\,x\,p_{i}\times C\,o\,e_{i}\right)$$

where, *N* is the number of prognostic genes, *Exp* is the gene expression value, and *Coe* is the estimated regression coefficient of the gene. A risk score for each patient was calculated by including the expression values of each selected gene, weighed by their estimated regression coefficients in the multivariate Cox regression analysis. The patients were divided into high- and low-risk groups using the median of the risk scores as the threshold. The receiver operating characteristic (ROC) curves were used to compute the sensitivity and specificity of overall prediction of the selected gene expression-based overall risk scores (ORSs) using the R package “survivalROC.” The area under curve (AUC) value was also calculated. The Kaplan–Meier method was applied to generate OS curves, and the log-rank test was used to assess the differences in OS between the high- and low-risk groups using the R package “survival.” Additionally, univariate and multivariate Cox proportional hazards regression, and data stratification analyses were performed to test whether the ORS was independent of other clinical features. Statistical significance was based on *p*-value < 0.05 and 95% confidence interval (CI) estimates.

To evaluate the performance of the risk model prediction, we randomly chose samples from the high- and low-risk group and trained a support vector machine (SVM) classifier based on the expression level of the selected genes in the risk model using the R package “e1071.” The 10-fold cross-validation method was used to evaluate the performance of the classifier. Plots of the ROC curve of the classifier and the calculation of the AUC were fulfilled using the R verification package. In addition, Chi-square test and Wilcoxon rank-sum test were also used in the study, and a *p*-value < 0.05 was considered as statistically significant. In differential expression analysis, the genes with the cutoff of *p*-value < 0.05 and logFC > 1 or < −1 between the two groups were regarded as statistically significant. All statistical analyses were performed using R version 3.6.3.

## Results

### Identification of Genome Instability-Related Genes in TNBC

**FIGURE 1:**
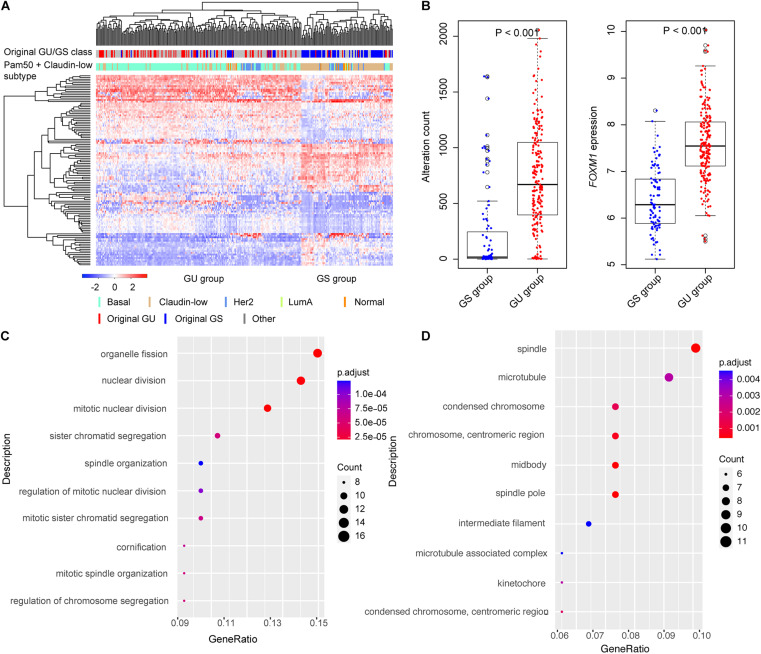
Identification of genome instability-related genes in triple-negative breast cancer (TNBC). **(A)** Hierarchical clustering of all the 299 Molecular Taxonomy of Breast Cancer International Consortium (METABRIC) TNBC cases using the expression of 111-genomic instability-related genes. The patients were divided into genomic unstable (GU) and genomic stable (GS) groups. **(B)** Boxplots of alteration count and *FOXM1* expression in GU and GS groups. The alteration counts and *FOXM1* expression in GU group were significantly higher than that in GS group. **(C)** The top 10 Gene Ontology Biological Process (GOBP) terms of functional enrichment results. **(D)** The top 10 Gene Ontology Cellular Component (GOCC) terms of functional enrichment results.

We identified 299 TNBC samples from the METABRIC breast cancer dataset and performed a systematic analysis (Supplementary Figure 1). To identify the genes associated with genomic instability, we calculated the cumulative count of alterations including somatic mutations and CNVs in each patient and sorted these counts in decreased order. The top 25% of TNBC patients (*n* = 75) were named as the GU group and the bottom 25% (*n* = 75) as the GS group. We performed differential expression analysis between the GU and GS groups. We identified 111 differentially expressed genes between the two groups, 63 upregulated and 48 downregulated in the GU group (Supplementary Table 1). We performed unsupervised hierarchical clustering analysis for all 299 TNBC samples by the 111 genes and compared our GU/GS groups with the PAM50 and claudin-low subtype available from the METABRIC dataset. The results showed that 69.1% (143/207) of the samples in the GU group were classified as basal subtype and 65.2% (60/92) samples in the GS group as the claudin-low subtype (Figure 1A). We found that all patients were classified into either the GU group or the GS group, in which the cumulative alterations in the GU group were significantly higher than that of the GS group (Figure 1B, Wilcox test *p* < 0.001). We further compared the expression level of *FOXM1*, a genome instability-related driver gene (Teh et al., 2010; Kim et al., 2013; Senfter et al., 2019), between the GU and GS groups. We found that the expression of *FOXM1* in the GU group was significantly higher than that in the GS group (Figure 1B, Wilcox test *p* < 0.001) and observed the expression of the classical proliferation gene *MKI67* was significantly higher in the GU group than that in the GS group (Supplementary Figure 2A, Wilcox test *p* < 0.001). To validate our defined GU/GS groups, we performed clustering analysis for the 111 genes in the 235 Shanghai TNBC samples and found that all samples were also significantly classified into the GU (191/235, 81.3%) and GS (44/235, 18.7%) groups (Supplementary Figure 3A). Besides, we compared the homologous recombination deficiency (HRD) levels between the GU/GS groups in the Shanghai dataset and observed that HRD level in the GU group was significantly higher than that in the GS group (Supplementary Figure 3B). We performed the same analysis using top 10% and bottom 10% as the cutoff for the GU/GS groups and received similar results from using the top 25% and bottom 25% as the cutoff (Supplementary Figure 2B).

To test whether these 111 differentially expressed genes were involved in important biological processes and pathways associated with genome instability, we performed functional enrichment analysis using R package “clusterProfiler” with BH adjustment for multiple testing. We identified multiple pathways associated with genomic instability, such as mitotic nuclear division, organelle fission, nuclear division, sister chromatid segregation, regulation of chromosome segregation, etc. (Figures 1C,D). We also performed DAVID analysis using the 111 genes. The results also showed that these genes were largely involved in cell cycle process, cell proliferation, and immune response (Supplementary Figure 4A), and some genes (such as *SFRP4*, *PRKCB*, *FZD9*, and *RAC2*) were known in involving TNBC development (Supplementary Figure 4B). The results highlight that the 111 differentially expressed genes were involved in tumorigenesis and development process of TNBC.

### Identification of GIGenSig for Prognostic Prediction

To explore the potential prognostic value of the above genome instability-related genes, we divided all the METABRIC TNBC cases into two subsets, the training dataset (*n* = 150) and the testing dataset (*n* = 149). To identify prognostic-associated genes, we conducted univariate Cox regression analysis to calculate the relationship between the 111 gene expression and OS in the training dataset. The result showed that 11 of the genes were associated with OS in TNBC (Table 2, *p* < 0.05). We named these 11 genes as GIGenSig. To evaluate the prognostic potential of GIGenSig, we constructed a prognostic risk model for OS based on the expression of GIGenSig and coefficients of multivariate Cox analysis: ORS = (0.137 × *PRKCB* expression) + (0.037 × *TFF3* expression) + (−0.008 × *ART3* expression) + (−0.071 × *CD52* expression) + (−0.030 × *CD79A* expression) + (−0.155 × *FZD9* expression) + (−0.010 × *GABRP* expression) + (−0.145 × *IRF8* expression) + (−0.187 × *ITM2A* expression) + (−0.038 × *SOX10* expression) + (−0.072 × *VGLL1* expression). Among the GIGenSig, the coefficients of *PRKCB* and *TFF3* were positive, suggesting that they were risk factors for TNBC, and their high expression was associated with poor survival of TNBC. In contrast, the coefficients for the other nine genes (*ART3*, *CD52*, *CD79A*, *FZD9*, *GABRP*, *IRF8 ITM2A*, *SOX10*, and *VGLL1*) were negative, suggesting that these were protective factors, and their higher expression was associated with better survival.

**TABLE 2:** Univariate Cox regression analysis for the 11 of 111-genome instability-related genes associated with overall survival in TNBC.

**Gene**	**Genomic location**	**Coefficient**	**HR**	**95% CI**	***p*-Value**
*ART3*	chr4:76932337–77033955	−0.126	0.882	0.778–0.999	0.048
*CD52*	chr1:26644448–26647014	−0.171	0.843	0.715–0.993	0.042
*CD79A*	chr19:42381190–42385439	−0.150	0.860	0.751–0.986	0.031
*FZD9*	chr7:72848109–72850450	−0.187	0.829	0.689–0.999	0.049
*GABRP*	chr5:170190354–170241051	−0.115	0.891	0.817–0.971	0.009
*IRF8*	chr16:85932409–85956215	−0.228	0.796	0.646–0.982	0.033
*ITM2A*	chrX:78615881–78623164	−0.188	0.829	0.690–0.997	0.046
*PRKCB*	chr16:23847322–24231932	−0.212	0.809	0.657–0.996	0.045
*SOX10*	chr22:38366693–38383429	−0.154	0.857	0.755–0.973	0.017
*TFF3*	chr21:43731777–43735761	0.106	1.112	1.011–1.224	0.029
*VGLL1*	chrX:135614311–135638966	−0.160	0.852	0.739–0.983	0.028

**FIGURE 2:**
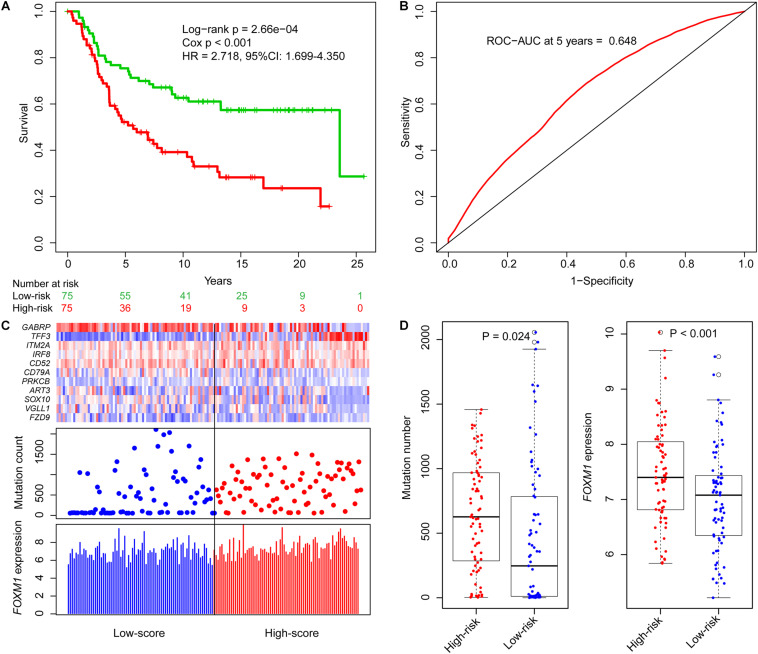
Identification of genome instability-derived gene signature (GIGenSig) for prognostic prediction. **(A)** Survival curve of overall survival of TNBC patients in the training dataset. Patients were significantly classified into high- and low-risk groups; **(B)** 5-year receiver operating characteristic (ROC) curve for the GIGenSig in the training dataset; **(C)** GIGenSig gene expression pattern and alteration distribution and *FOXM1* expression level with the increasing overall risk score (ORS) scores for the patients in the training dataset. The blue and red represent the low- and high-risk groups, respectively; **(D)** distribution of accumulative alteration number and *FOXM1* expression in the high- and low-risk groups in the training dataset. The blue and red represent the low- and high-risk groups, respectively.

Based on ORS values, TNBC patients in the training datasets were classified into two groups by their median ORS (−4.602), named as high-risk group and low-risk group. Survival plot showed that the OS survival in the low-risk group was significantly better than those of the high-risk group (Figure 2A, log-rank test *p* = 2.66e−04; HR = 2.718, 95% CI: 1.699–4.350), the 5-year survival rate in the low-risk group (73%) was higher than that in high-risk group (48%), and the 5-year ROC curve analysis provided an AUC of 0.648 (Figure 2B). In addition, SVM and 10-fold cross-validation showed that our risk model was robust to classify TNBC patients into high- and low-risk groups (Supplementary Figure 5A, AUC = 0.987, *p* = 7.62e−33).

We ranked the ORS for patients in the training dataset to explore the differences of GIGenSig expression, alteration count, and *FOXM1* expression between low score and high score groups (Figure 2C). Clustering analysis showed that *PRKCB* and *TFF3* were upregulated in the high score group, whereas the other nine genes were upregulated in the low score group (Figure 2C). The differences of alteration count and *FOXM1* expression were both significant between the high-risk and low-risk groups (Figure 2D). The count of alterations in the high-risk group was significantly higher than that in the low-risk group (Figure 2D, Wilcoxon test *p* = 0.024). Additionally, *FOXM1* had significantly higher expression in the high-risk group than in the low-risk group (Figure 2D, Wilcoxon test *p* < 0.001).

### Validation of GIGenSig for Prognostic Prediction in Testing and Molecular Taxonomy of Breast Cancer International Consortium Datasets

**FIGURE 3:**
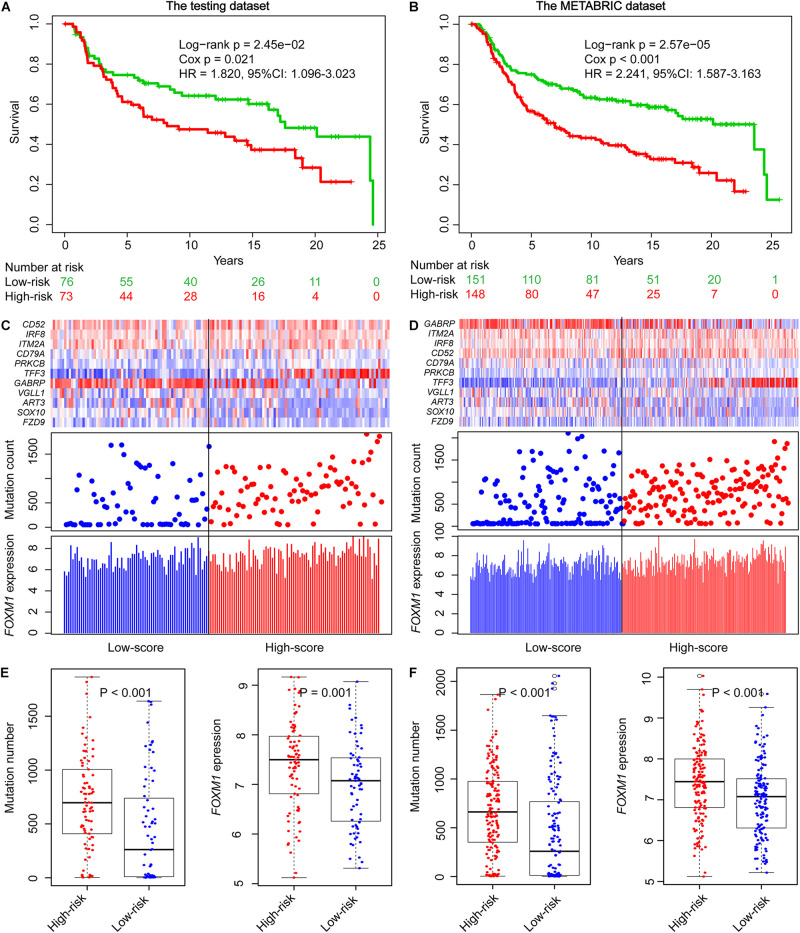
Validation of GIGenSig for prognostic prediction in the testing and METABRIC datasets. **(A,B)** Survival curves of overall survival of patients in the testing and METABRIC datasets. Patients were significantly classified into high- and low-risk groups. **(C,D)** The GIGenSig gene expression pattern, alteration distribution, and *FOXM1* expression level with the increasing ORS scores for the patients in the testing and METABRIC dataset. The blue and red represent the low- and high-risk groups, respectively. **(E,F)** The distribution of accumulative alteration number and *FOXM1* expression in the high- and low-risk groups in the testing and METABRIC datasets. The blue and red represent the low- and high-risk groups, respectively.

To explore the prognostic performance of GIGenSig, we tested it using the testing dataset of 149 TNBC cases. Based on the ORS cutoff in the training dataset, the cases in the testing dataset were classified into high-risk group (*n* = 73) and low-risk group (*n* = 76). The OS of the low-risk group was significantly higher than that of the high-risk group (Figure 3A, log-rank test *p* = 2.45e−02; HR = 1.820, 95% CI: 1.099–3.023). Similarly, the 5-year survival rate in the low-risk group (72%) was also higher than that in the high-risk group (60%), and the 5-year ROC analysis yielded an AUC of 0.607 (Supplementary Figure 6A). Additionally, the SVM and 10-fold cross-validation showed that our risk model was robust to classify TNBC patients into high- and low-risk groups in the testing dataset (Supplementary Figure 5B, AUC = 0.980, *p* = 8.64e−32). We also displayed the clustering of GIGenSig, alteration count, and *FOXM1* expression level according to the increasing order of the ORS for each patient in the testing dataset (Figure 3C). The alteration count and *FOXM1* expression level were both significantly higher in the high-risk group than in the low-risk group (Figure 3E, Wilcoxon test *p* < 0.001 for alteration count; Wilcoxon test *p* = 0.001 for *FOXM1* expression level).

We also validated the prognostic power of GIGenSig in the METABRIC TNBC dataset. The patients were classified into two groups. The median survival time of the low-risk group was significantly higher than that of the high-risk group (Figure 3B, median: 23.6 vs. 7 years; log-rank test *p* = 2.57e−05; HR = 2.241, 95% CI: 1.587–3.63). The 5-year survival rate in the low-risk group was longer (72%) than that in the high-risk group (54%), and the 5-year ROC gave an AUC of 0.627 (Supplementary Figure 6B). In addition, the SVM and 10-fold cross-validation showed that our risk model was robust to classify TNBC patients into high- and low-risk groups in the METABRIC dataset (Supplementary Figure 5C, AUC = 0.980, *p* = 5.30e−62). A similar pattern was observed in the METABRIC dataset as in the training and testing datasets for the clustering of GIGenSig, alteration count, and *FOXM1* expression (Figure 3D). Additionally, both significant differences were present for alteration count and *FOXM1* expression between the high-risk and low-risk groups (Figure 3F, Wilcoxon test *p* < 0.001 for alteration count; Wilcoxon test *p* < 0.001 for *FOXM1* expression level).

### Validation of GIGenSig in Five Additional Datasets

**FIGURE 4:**
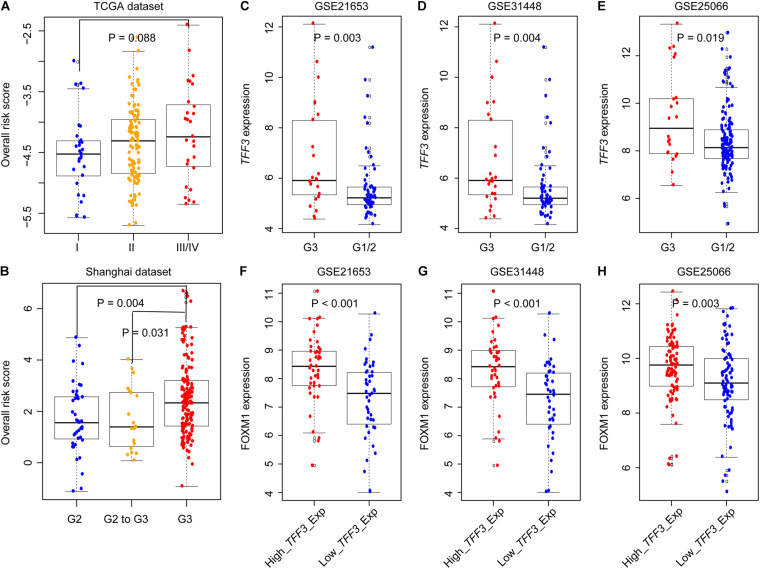
Validation of GIGenSig in five additional datasets. **(A,B)** Boxplots for ORS for TNBC patients with different stage and grade in The Cancer Genome Atlas (TCGA) and Shanghai dataset. **(C–E)** Boxplots for *TFF3* expression among patients with different grade in GSE21653, GSE31448, and GSE25066. **(F–H)** Boxplots for *FOXM1* expression among patients with high and low *TFF3* expression in GSE21653, GSE31448, and GSE25066. The comparisons between any two different groups were performed by Wilcox test.

We compared GIGenSig using two independent datasets, the TCGA and Shanghai TNBC data, to test if clinical stage and grade could have an impact on the prognosis of TNBC. As shown in Figure 4A, there was a close relationship between the stage of TNBC and ORS but not reaching a significant level in the TCGA dataset (Figure 4A, Wilcoxon test *p* = 0.088). ORS was also associated with TNBC grade in Shanghai dataset that ORS of grade 3 was significantly higher than that of grade 2 and grades 2–3 (Figure 4B, *p* = 0.004 for comparing with grade 2; *p* = 0.031 for comparing with grades 2–3; Wilcoxon test). We further validated GIGenSig in another three independent breast cancer datasets generated by the microarray platform (GSE21653, GSE31448, and GSE25066). We reannotated the microarray data to obtain the gene expression data and extracted the common clinical characteristics from the three datasets. We then examined the association of GIGenSig with TNBC genomic instability information in these three independent datasets. Among all the 111 genes in GIGenSig, we found that *TFF3* presented significantly higher expression level in grade 3 than in grades 1 and 2 in all three datasets (Figures 4C–E, *p* = 0.003 for GSE21653; *p* = 0.004 for GSE31448; and *p* = 0.019 for GSE25066; Wilcoxon test). Furthermore, we tested the relationship between *TFF3* expression and *FOXM1* expression in the three datasets. We observed that *FOXM1* expression in patients with high *TFF3* expression was significantly higher than that with low *TFF3* expression in all three datasets (Figures 4F–H, *p* < 0.001 for GSE21653; *p* < 0.001 for GSE31448; and *p* = 0.003 for GSE25066; Wilcoxon test).

### Prognostic Prediction by GIGenSig Is Independent of Clinical Features

**FIGURE 5:**
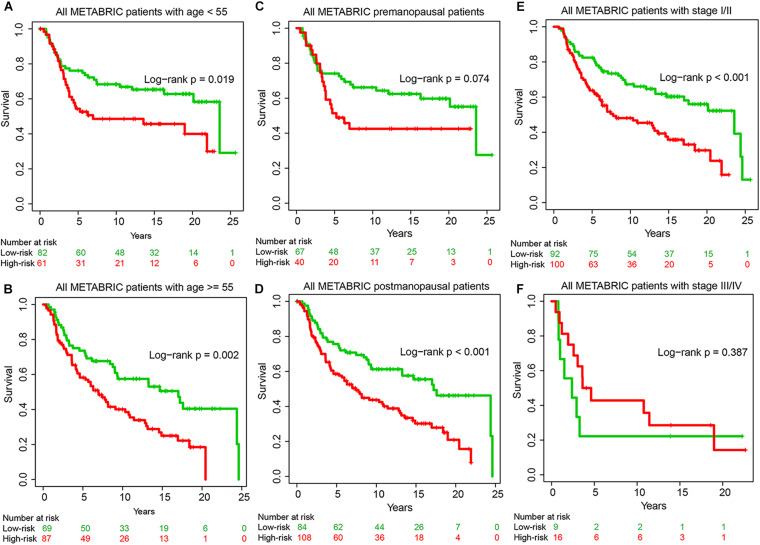
Prognostic prediction by GIGenSig is independent of clinical features. **(A,B)** Survival curve of overall survival (OS) for patients with age <55 and ≥55 in the METABRIC datasets. Patients were significantly classified into high- and low-risk groups. **(C,D)** Survival curve of OS for patients with premenopausal and postmenopausal status in the METABRIC datasets. Patients were classified into high- and low-risk groups; **(E,F)** Survival curve of OS for patients with stages I and II and stages III and IV in the METABRIC datasets. Patients were classified into high- and low-risk groups.

To explore whether the prognostic ability of GIGenSig was independent of age, menopausal status, and tumor stage, we performed univariate and multivariate Cox regression analysis. The results showed that the GIGenSig was significantly associated with TNBC OS in the three datasets when adjusted by age, menopausal status, tumor stage, and grade (Table 3). The METABRIC patients were divided into two groups according to age with <55 and ≥55, pre- and post-status of menopause, and tumor stage with I/II and III/IV. The patients were classified into high- and low-risk groups according to the median risk scores in the training dataset. The results revealed that the patients were significantly classified into two groups by age (Figures 5A,B; log-rank test *p* = 0.019 for age < 55 group; log-rank test *p* = 0.002 for age ≥ 55 group), menopausal (Figures 5C,D; log-rank test *p* = 0.074 for premenopausal group; log-rank test *p* < 0.001 for postmenopausal group), and stage subset (Figures 5E,F; log-rank test *p* < 0.001 for stage I/II group; log-rank test *p* = 0.387 for stage III/IV group). The classification for patients in the stage III/IV group was not significant probably due to the smaller sample size (*n* = 25) in this group. These results indicated that GIGenSig served as a prognostic signature for TNBC independent of age, menopausal status, and tumor stage.

**TABLE 3:** Univariate and multivariate Cox regression analyses of the genome instability-derived gene signature (GIGenSig) and overall survival in different patient datasets.

**Characteristics**	**Univariable analysis**			**Multivariable analysis**		
	**HR**	**95% CI**	***p*-value**	**HR**	**95% CI**	***p*-Value**
Training dataset						
GIGenSig	2.718	1.699–4.350	<0.001	2.184	1.218–3.915	0.009
Age	1.022	1.004–1.041	0.019	1.042	1.007–1.079	0.018
Menopausal status	0.887	0.549–1.433	0.625	2.656	1.024–6.886	0.045
Tumor stage	1.358	0.691–2.672	0.375	0.963	0.458–2.024	0.921
Grade	1.187	0.593–2.375	0.629	1.870	0.822–4.255	0.135
Testing dataset						
GIGenSig	1.820	1.096–3.023	0.021	1.354	0.683–2.682	0.038
Age	1.024	1.008–1.040	0.004	1.035	1.000–1.072	0.052
Menopausal status	0.640	0.395–1.037	0.070	2.215	0.754–6.508	0.148
Tumor stage	4.682	2.255–9.721	<0.001	4.976	2.266–10.926	<0.001
Grade	0.899	0.504–1.605	0.719	1.485	0.689–3.203	0.313
METABRIC dataset						
GIGenSig	2.241	1.587–3.163	<0.001	1.647	1.080–2.512	0.020
Age	1.024	1.012–1.036	0.000	1.037	1.013–1.062	0.002
Menopausal status	0.736	0.525–1.031	0.075	2.270	1.137–4.531	0.020
Tumor stage	2.180	1.332–3.569	0.002	1.851	1.093–3.134	0.022
Grade	1.011	0.649–1.573	0.962	1.456	0.842–2.515	0.178

### Genome Instability-Derived Gene Signature Performs Better Than Other Prognostic Signatures

**FIGURE 6:**
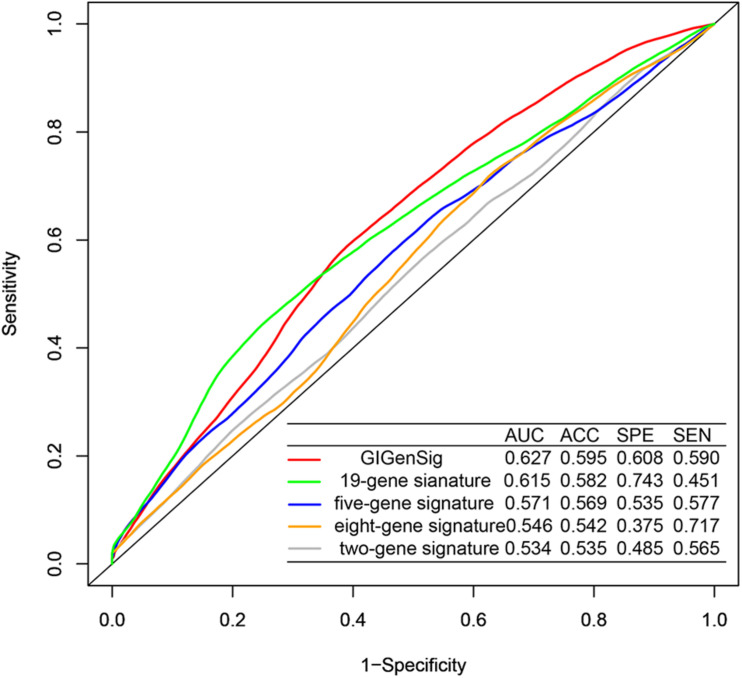
Better performance of GIGenSig than other prognostic signatures. Five-year ROC comparison of overall survival between GIGenSig signature and other four signatures from AUC (area under the curve), ACC (accuracy), SPE (specificity), and SEN (sensitivity).

To further explore the prognostic performance of the GIGenSig, we compared GIGenSig with other TNBC prognostic signatures including the two-gene signature (Alsaleem et al., 2020), the five-gene signature (Wang et al., 2018), the eight-gene signature (Kim et al., 2019), and the 19-gene signature (Qian et al., 2017) using the METABRIC dataset. The result showed that the 5-year AUC (0.627) of OS for GIGenSig was significantly higher than that of the two-gene signature (AUC = 0.534), the five-gene signature (AUC = 0.571), the eight-gene signature (AUC = 0.546), and the 19-gene signature (AUC = 0.615) (Figure 6). The results demonstrated that the GIGenSig provided better prognostic prediction for TNBC than the other four signatures.

## Discussion

Compared with TNBC treatment, limited progress has been made in TNBC prognosis (Sulaiman et al., 2018a,b; Mou and Wang, 2019; Zhang et al., 2019). Prognostic study usually evaluates clinical features, such as tumor size, stage, grade, etc., which provide limited mechanistic information to understand the relationship between prognosis and the disease (Echavarria et al., 2018; Johansson et al., 2018; Park et al., 2019). Genome instability and abnormal gene expression are common features in cancer (Telli et al., 2016; Tutt et al., 2018; Huang et al., 2019). While the relationship between genome instability and dysregulation of gene expression in cancer has been studied, genome-wide characterization for its prognostic value in TNBC has not been systematically analyzed (Grady and Carethers, 2008; Rao et al., 2017; Kalimutho et al., 2019).

As shown from our current study, molecular evidence from genome instability and abnormal gene expression is a rich resource to identify prognostic signatures as the prognostic marker for TNBC. In our study, we identified 111 genome instability-related genes by integrating mutation, CNV, and gene expression from TNBC. Functional analysis showed that these 111 genomic instability-related genes were enriched in the pathways associated with mitotic process. Dysregulation of mitotic processes can impact DNA replication involving mitotic nuclear division, nuclear division, and organelle fission, contributing to genome instability and OS of TNBC (Chen L. et al., 2018; Si et al., 2020; Suo et al., 2020). For example, the feedback loop between Drp1-mediated mitochondrial fission and Notch signaling pathway can promote TNBC cell survival via increasing survivin expression (Chen L. et al., 2018), and silibinin-induced mitochondrial fission can cause mitophagy preventing silibinin-induced apoptosis in TNBC (Si et al., 2020). Functional annotation with DAVID tool revealed that these 111-genomic instability-related genes play important roles in carcinogenetic pathways, such as affecting cell cycle (Kastan and Bartek, 2004), uncontrolled cell proliferation (Evan and Vousden, 2001), and abnormal immune response (Desrichard et al., 2016).

From the 111 genes, we further identified a GIGenSig with 11 genes (*ART3*, *CD52*, *CD79A*, *FZD9*, *GABRP*, *IRF8*, *ITM2A*, *PRKCB*, *SOX10*, *TFF3*, and *VGLL1*). Our study demonstrated that the GIGenSig effectively divided TNBC into high- and low-risk groups in the training dataset and its prognostic value was validated independently in multiple testing datasets. Besides, GIGenSig was significantly associated with genomic alteration pattern and *FOXM1* expression, which are important predictors of genome instability. Certain genes in GIGenSig are known to be closely related to tumorigenesis and development of TNBC. For example, *ART3* overexpression regulated TNBC cell functions by activating AKT and ERK pathways (Tan et al., 2016); *GABRP* and *VGLL1* were present in basal-like/triple-negative phenotype, and their expression levels were associated with OS of TNBC (Castilla et al., 2014; Sizemore et al., 2014); and *ITM2A* and *SOX10* were prognostic biomarkers for TNBC and potential therapeutic targets (Harbhajanka et al., 2018; Abuderman et al., 2020).

Furthermore, our identified GIGenSig can have clinical significance in TNBC treatment. It has been reported that Ki67 encoded by *MKI67* plays an important role in the prognosis and treatment of breast cancer (Yerushalmi et al., 2010; Li et al., 2015; Jurikova et al., 2016). Using the METABRIC dataset, we also observed that older TNBC patients had significantly lower *MKI67* expression than younger TNBC patients (Supplementary Figure 7A, Wilcox test *p* = 2.59e−3). However, Ki67 did not show prognostic value for patients used in our study (Supplementary Figure 7B). This implies that a combination of multiple genes provides better prognostic power than does a single gene. Our study also found that TNBC patients with chemotherapy treatment had low risk (84 vs. 73 with chemotherapy in the low-risk and high-risk groups, Supplementary Figure 7C), demonstrating the effectiveness of chemotherapy as the main therapeutic strategy in TNBC treatment (Denkert et al., 2017; Chaudhary et al., 2018; Lyons, 2019).

In conclusion, our study provides a genome instability-based TNBC prognostic signature to predict the clinical outcome of TNBC. Further tests with more datasets and clinical information will validate its value for clinical TNBC applications.

## Data Availability Statement

Publicly available datasets were analyzed in this study. These are included in the article/Supplementary Material.

## Author Contributions

MG and SW conceptualized the study and reviewed and edited the manuscript. MG formulated the methodology, performed the investigation and visualization, and wrote the original draft. SW supervised and secured the funding. Both authors contributed to the article and approved the submitted version.

## Conflict of Interest

The authors declare that the research was conducted in the absence of any commercial or financial relationships that could be construed as a potential conflict of interest.

## Footnotes

1 https://www.cbioportal.org/

2 https://www.gencodegenes.org

3 http://xena.ucsc.edu/

4 https://www.ncbi.nlm.nih.gov/sra/?term=SRP157974

5 https://www.ncbi.nlm.nih.gov/geo/query/acc.cgi?acc=GSE21653

6 https://www.ncbi.nlm.nih.gov/geo/query/acc.cgi?acc=GSE31448

7 https://www.ncbi.nlm.nih.gov/geo/query/acc.cgi?acc=GSE25066

8 http://www.affymetrix.com

9 http://david.abcc.ncifcrf.gov/
