# Supplementary material for: Genome Instability-Derived Genes Are Novel Prognostic Biomarkers for Triple-Negative Breast Cancer
Source: Front Cell Dev Biol. 2021 Jul 12;9:701073. doi: 10.3389/fcell.2021.701073 (PMC8312551; doi:10.3389/fcell.2021.701073)
Supplement: Supplementary file 1 [file Data_Sheet_1.docx]

**Supplementary** **Figures and Tables**

**Supplementary** **Figure Legends**

**Figure S1.** Flowchart of the analytical process. Genome instability-related genes were identified by integrating somatic mutations and CNVs, and gene expression profiles in TNBC cases of the METABRIC dataset. A genome instability-derived gene signature (GIGenSig) of 11 genes was identified by univariate Cox proportional hazards regression analysis as a prognostic biomarker for TNBC. See detailed description in the text.

**Figure S2.** Related to Figure 1. (A) Boxplots of *FOXM1*, *MKI67* expression in GU and GS groups. The *FOXM1* and *MKI67* expression in GU group was significantly higher than that in GS group. (B) Hierarchical clustering of all the 299 METABRIC TNBC cases using the expression of 383 genomic instability-related genes identified by comparing top 10% and bottom 10% patients. The patients were divided into GU and GS groups.

**Figure S3.** Related to Figure 1. Identification of genome instability-related genes in TNBC. (A) Hierarchical-clustering of all the 235 Shanghai TNBC cases using the expression of 111 genome instability-related genes. All the patients were divided into GU or GS groups. (B) Boxplots of HRD scores in GU and GS groups. The HRD scores in GU group is significantly higher than that in GS group.

**Figure S4.** Related to Figure 1. Functional enrichment analysis for 111 genome instability-related genes in TNBC. The barplot for the top 30 significant GO functional enriched biological processes (BPs). (B) Wnt signaling pathway. The marked rings indicate the locations of genome instability-related genes involved in this pathway.

**Figure S5.** Related to Figure 2 and 3. The risk model predictive performance evaluation based on expression values of 111 genome instability-related genes assigns all samples into high- and low-risk groups with high accuracy, with AUC of 0.987 in the training dataset, 0.980 in the testing dataset and 0.980 in the METABRIC dataset.

**Figure S6**. Related to Figure 3. Validation of GIGenSig for prognostic prediction in the testing and METABRIC datasets. (A) The 5-year ROC curve for the GIGenSig in the testing dataset. (B) The 5-year ROC curve for the GIGenSig in the METABRIC dataset.

**Figure S7.** (A) Boxplot for *MKI67* expression for TNBC patients with different age groups in the METABRIC dataset. (B) Survival curve of overall survival of TNBC patients in the METABRIC using *MKI67* expression as predictor. (C) Barplot for the patients with or without chemotherapy in high- and low-risk groups in the METABRIC dataset.


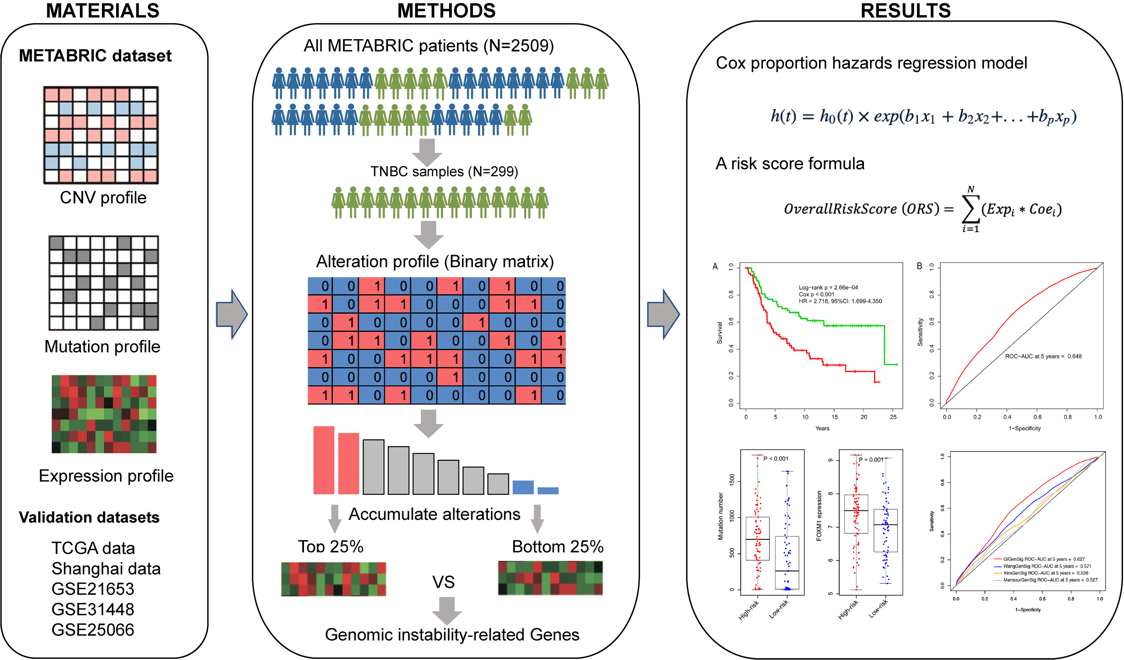


**Figure S1**


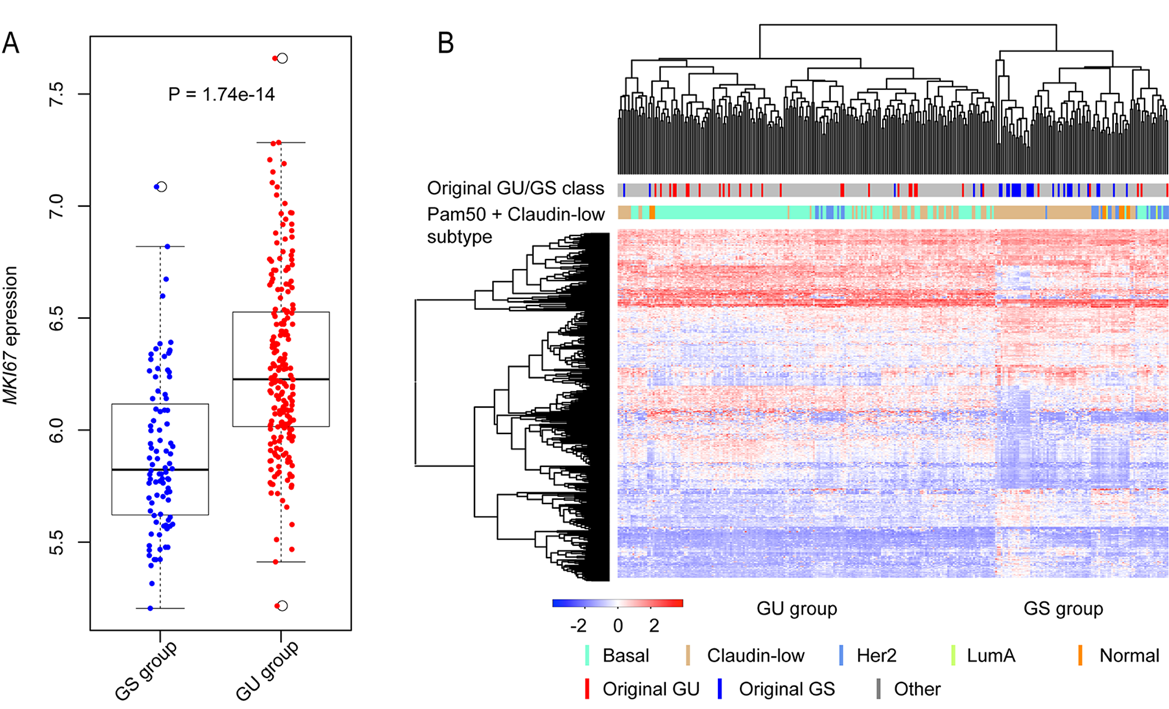


**Figure S2**


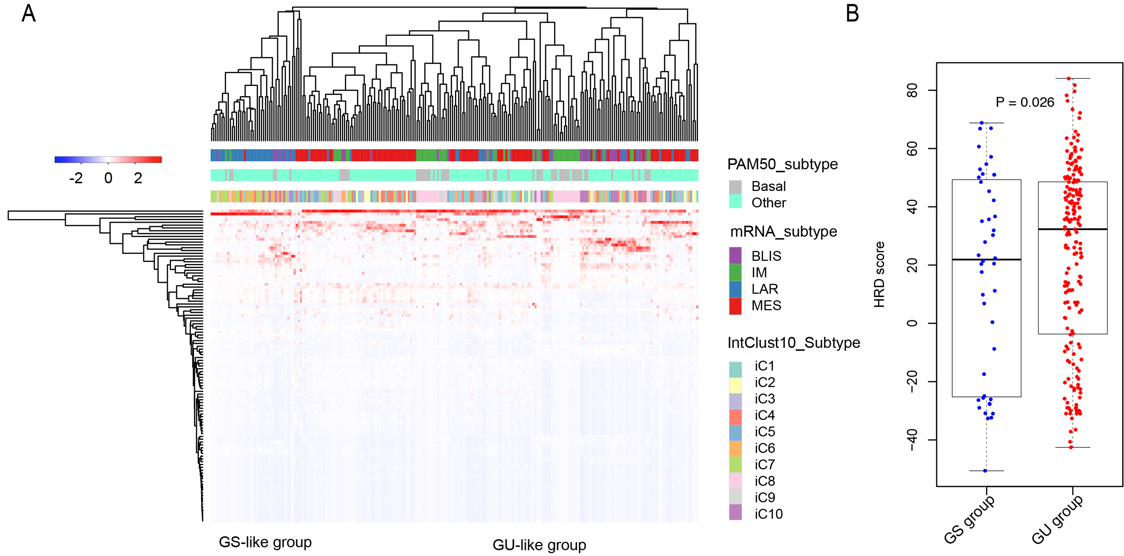


**Figure S3**


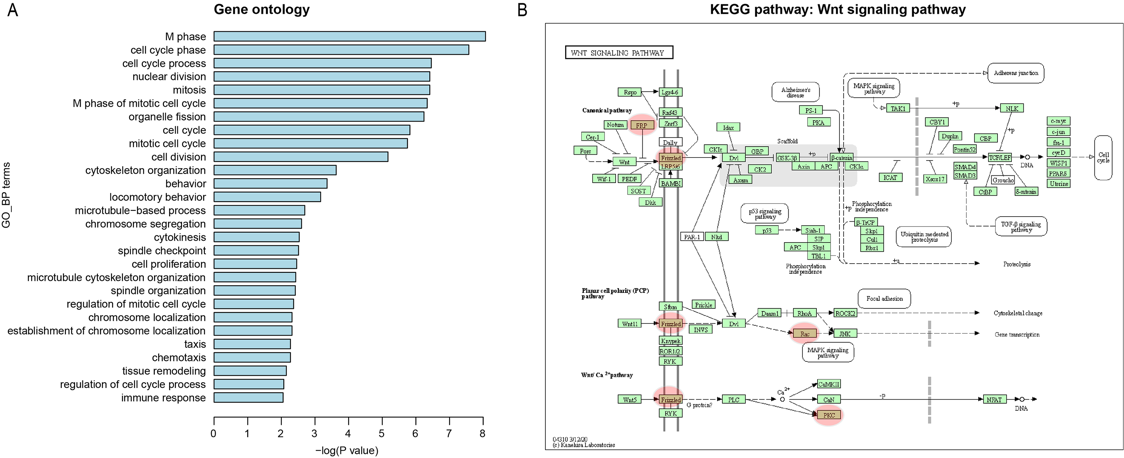


**Figure S4**


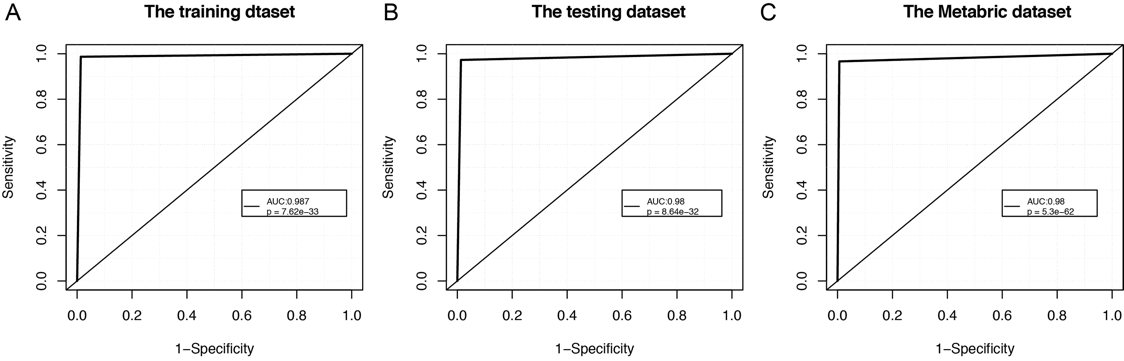


**Figure S5**

**
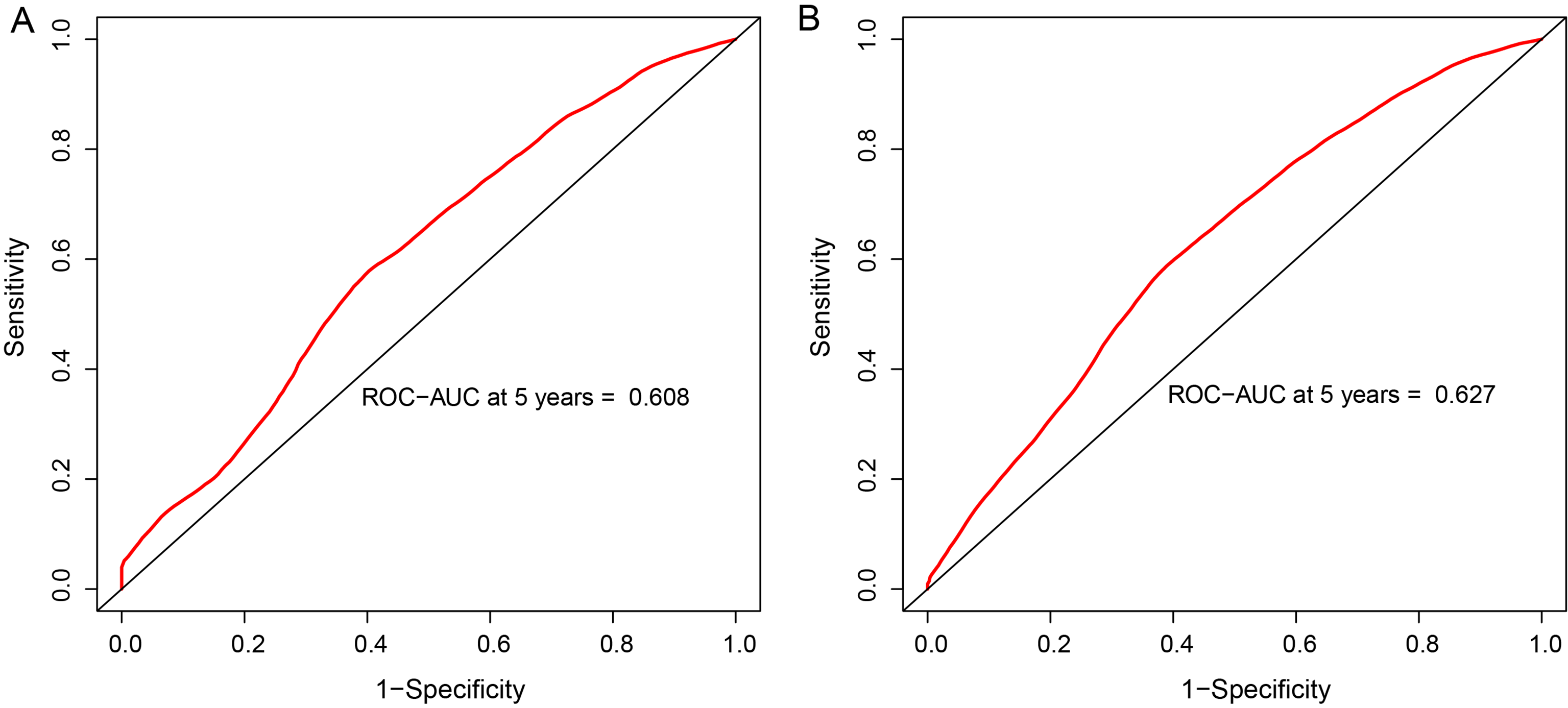
**

**Figure S6**


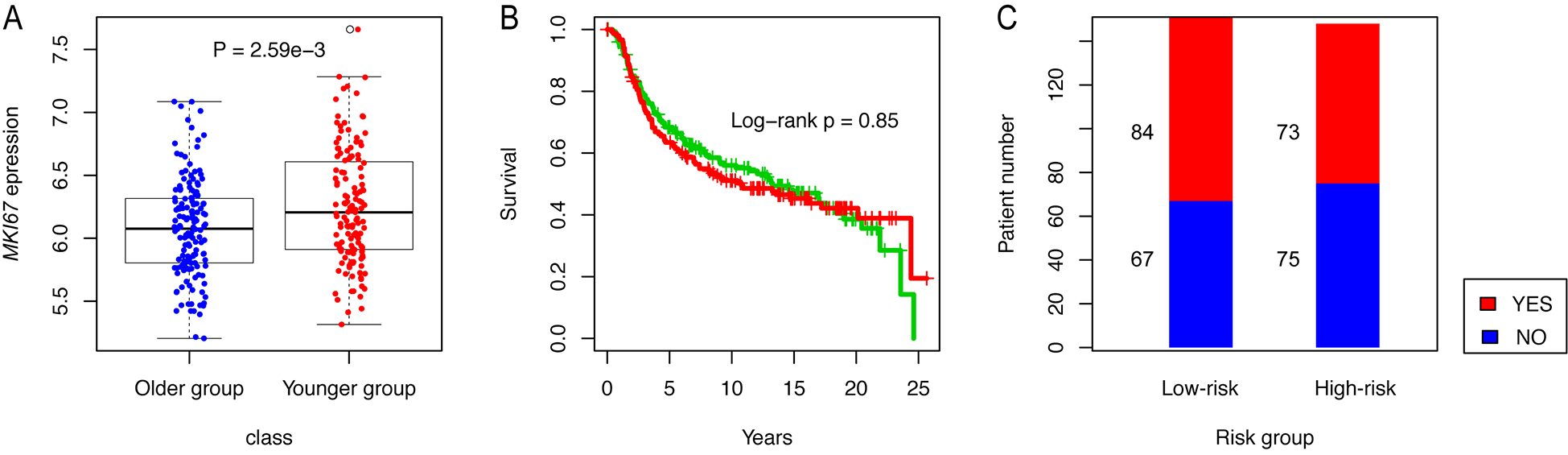


**Figure S7**

**Supplementary Table S1.** List of 111 genome instability-related genes in TNBC

| **GeneName** | **P-value** | **LogFC** |
| --- | --- | --- |
| *ABI3BP* | 1.50E-12 | -1.060 |
| *ADH1A* | 2.46E-06 | -1.187 |
| *ALDH1A1* | 4.72E-11 | -1.027 |
| *ANKRD30A* | 3.53E-04 | -1.048 |
| *APOD* | 5.28E-07 | -1.669 |
| *ART3* | 2.95E-05 | 1.399 |
| *ASPM* | 2.13E-14 | 1.171 |
| *AURKA* | 8.75E-14 | 1.015 |
| *AURKB* | 1.64E-14 | 1.085 |
| *BAIAP2L1* | 6.30E-12 | 1.021 |
| *BIRC5* | 5.58E-10 | 1.057 |
| *C1orf106* | 6.36E-08 | 1.085 |
| *CBX2* | 7.94E-12 | 1.235 |
| *CCL15* | 7.86E-10 | -1.317 |
| *CCL19* | 3.22E-05 | -1.302 |
| *CCL21* | 5.14E-06 | -1.134 |
| *CCNB2* | 3.15E-12 | 1.183 |
| *CD52* | 6.79E-07 | -1.288 |
| *CD79A* | 1.74E-04 | -1.069 |
| *CDC20* | 3.05E-14 | 1.527 |
| *CDCA5* | 2.79E-12 | 1.105 |
| *CDCA7* | 6.22E-09 | 1.057 |
| *CENPF* | 9.37E-15 | 1.177 |
| *CFD* | 1.96E-08 | -1.163 |
| *CGN* | 2.58E-08 | 1.019 |
| *CITED4* | 1.25E-08 | 1.053 |
| *CKS1B* | 3.21E-09 | 1.064 |
| *CLDN5* | 9.58E-12 | -1.017 |
| *COLEC12* | 8.44E-09 | -1.005 |
| *CTSG* | 2.32E-07 | -1.054 |
| *CTSK* | 1.81E-07 | -1.005 |
| *CXCL12* | 1.05E-11 | -1.363 |
| *DCN* | 2.19E-08 | -1.289 |
| *DHRS9* | 6.43E-10 | -1.193 |
| *DSP* | 3.22E-08 | 1.082 |
| *ELF3* | 1.48E-09 | 1.187 |
| *ELF5* | 3.92E-07 | 1.677 |
| *ENPP2* | 4.62E-10 | -1.071 |
| *EXO1* | 9.96E-14 | 1.045 |
| *FABP4* | 2.16E-06 | -1.257 |
| *FCER1A* | 5.59E-07 | -1.004 |
| *FOS* | 3.44E-05 | -1.011 |
| *FOXC1* | 2.37E-06 | 1.416 |
| *FOXM1* | 1.74E-12 | 1.071 |
| *FZD9* | 2.10E-08 | 1.088 |
| *GABRP* | 1.87E-04 | 1.589 |
| *GIMAP7* | 2.46E-08 | -1.008 |
| *GZMK* | 1.79E-06 | -1.121 |
| *HBA2* | 1.66E-07 | -1.364 |
| *HBB* | 7.83E-07 | -1.359 |
| *IRF8* | 1.40E-08 | -1.024 |
| *ITM2A* | 1.63E-12 | -1.470 |
| *KIF1A* | 2.83E-04 | 1.056 |
| *KIF20A* | 5.26E-15 | 1.066 |
| *KIF2C* | 4.52E-14 | 1.004 |
| *KLK5* | 3.07E-03 | 1.142 |
| *KLRB1* | 4.65E-09 | -1.053 |
| *KRT17* | 5.88E-04 | 1.059 |
| *KRT19* | 1.68E-05 | 1.246 |
| *KRT6B* | 2.55E-03 | 1.221 |
| *KRT7* | 3.32E-05 | 1.057 |
| *KRT81* | 1.01E-05 | 1.804 |
| *KRT86* | 8.38E-07 | 1.166 |
| *LAD1* | 3.18E-07 | 1.160 |
| *LCN2* | 1.09E-03 | 1.057 |
| *LYZ* | 2.21E-05 | -1.133 |
| *MCM10* | 7.62E-15 | 1.119 |
| *MELK* | 1.61E-12 | 1.180 |
| *MFAP4* | 2.70E-10 | -1.225 |
| *MSLN* | 9.16E-05 | 1.384 |
| *MT1G* | 4.10E-05 | 1.000 |
| *MUC16* | 8.83E-08 | 1.194 |
| *MYH11* | 2.75E-07 | -1.182 |
| *NDN* | 1.69E-11 | -1.048 |
| *NEK2* | 2.79E-14 | 1.142 |
| *PHGDH* | 5.13E-07 | 1.005 |
| *PIP* | 2.10E-02 | -1.041 |
| *PITX1* | 1.51E-09 | 1.532 |
| *PLAC9* | 2.38E-09 | -1.093 |
| *PRC1* | 1.02E-11 | 1.070 |
| *PRKCB* | 3.35E-08 | -1.066 |
| *PROM1* | 4.82E-07 | 1.661 |
| *PTGDS* | 1.99E-11 | -1.675 |
| *RAB25* | 2.07E-07 | 1.075 |
| *RAC2* | 3.54E-08 | -1.036 |
| *RASD2* | 9.08E-08 | 1.058 |
| *RHPN2* | 5.22E-08 | 1.064 |
| *ROPN1* | 1.89E-06 | 1.415 |
| *ROPN1B* | 1.68E-06 | 1.326 |
| *S100P* | 3.31E-04 | 1.271 |
| *SEPP1* | 3.77E-09 | -1.480 |
| *SFRP4* | 3.90E-06 | -1.134 |
| *SLC40A1* | 4.52E-13 | -1.343 |
| *SOX10* | 4.54E-04 | 1.012 |
| *SPARCL1* | 5.60E-08 | -1.012 |
| *SRPX* | 3.15E-07 | -1.005 |
| *SVEP1* | 1.24E-11 | -1.074 |
| *TFAP2C* | 3.37E-09 | 1.095 |
| *TFF3* | 4.68E-04 | -1.342 |
| *TGFBR2* | 8.84E-12 | -1.013 |
| *TMEM119* | 9.10E-10 | -1.043 |
| *TMSB15A* | 7.64E-06 | 1.066 |
| *TOP2A* | 1.35E-11 | 1.146 |
| *TPX2* | 3.55E-13 | 1.004 |
| *TRIP13* | 6.17E-14 | 1.151 |
| *TSPAN7* | 5.11E-09 | -1.036 |
| *TTK* | 3.56E-15 | 1.173 |
| *UBE2C* | 3.16E-12 | 1.469 |
| *UBE2T* | 1.70E-14 | 1.152 |
| *VGLL1* | 3.13E-05 | 1.077 |
| *VTCN1* | 2.59E-06 | 1.405 |
